# Effects of Biological Sex and Age on Cerebrospinal Fluid Markers—A Retrospective Observational Study

**DOI:** 10.1002/acn3.70307

**Published:** 2026-01-12

**Authors:** Isabel‐Sophie Hafer, Simon Faissner, Ralf Gold, Aiden Haghikia

**Affiliations:** ^1^ Department of Neurology Ruhr‐University Bochum, St Josef Hospital Bochum Germany; ^2^ Department of Neurology and Clinical Neurophysiology Medizinische Hochschule Hannover (MHH) Hannover Germany

**Keywords:** CSF markers, reference values, sex and age‐dependent CSF markers

## Abstract

**Objective:**

Cerebrospinal fluid (CSF) analysis is a key diagnostic tool for neurological diseases. To date, only a few studies have investigated in larger cohorts the effect of age and biological sex on diagnostic markers extracted from CSF.

**Methods:**

For this retrospective observational study, 4163 CSF findings (2012–2020) were evaluated. After exclusion of pathogenic CSF findings above or below the locally established reference ranges that are routinely applied in the CSF laboratory for clinical reporting, 1270 findings were included for statistical analysis. Using regression analysis, the relationship between age and CSF markers was illustrated, and regression equations were created.

**Results:**

A significant effect of sex was shown for CSF albumin (*p* < 0.001; Δ*M* 29.34), CSF protein (*p* < 0.001; Δ*M* 39.38) and CSF glucose (*p* < 0.001; Δ*M* 3.63) as well as CSF/serum albumin quotient (Q Alb) (*p* < 0.001; Δ*M* 0.57), for Immunoglobulin G in CSF (IgG CSF) (*p* < 0.001; Δ*M* 2.48), CSF/serum IgG quotient (Q IgG) (*p* < 0.001; Δ*M* 0.27), Immunoglobulin A in CSF (IgA CSF) (*p* < 0.001; Δ*M* 0.52), CSF/serum IgA quotient (Q IgA) (*p* < 0.001; Δ*M* 0.15) and CSF/serum IgM quotient (Q IgM) (*p* < 0.001; Δ*M* 0.09). Age showed a significant effect throughout most CSF markers, most strongly for CSF protein (*p* < 0.001; *η* ^2^ = 0.16) and Q Alb (*p* < 0.001; *η* ^2^ = 0.23). Both parameters increased significantly with age, leading to higher mean values in older individuals.

**Interpretation:**

For almost all markers in CSF, a significant effect of age alone on the markers or a significant effect of sex and age on the markers could be detected. Our study underscores the importance of age‐ and sex‐adjusted reference values for the interpretation of CSF markers in clinical practice.

## 1 Introduction

CSF analysis is a diagnostic procedure regularly performed in everyday clinical practice to detect and confirm neurological diseases [1, 2, 3, 4, 5]. CSF analysis is essential for the detection of an inflammatory or autoimmune process, the diagnosis of a neurodegenerative process such as Alzheimer's disease, the diagnosis of a neoplastic neurological disease as well as a computed tomography (CT) negative subarachnoid hemorrhage [2, 3, 5, 6]. Furthermore, it is used to assess the course and progression of neurological diseases and to monitor treatment responses [5, 7, 8]. Therapeutic applications of medications such as chemotherapy or small interfering ribonucleic acid (siRNA) complement the indication for lumbar punctures. Although lumbar puncture is performed at any age and sex, there are few studies having investigated the effect of age and sex on CSF parameters in larger cohorts with no neurological disease [5, 9, 10, 11, 12, 13]. The CSF diagnostic work‐up includes determination of cell count, protein, lactate and glucose in CSF which provides information on inflammatory changes, hemorrhage and presence of bacterial or viral infection in CSF [1]. The IgG Index is used to differentiate intrathecal IgG synthesis from a dysfunction of the blood–brain barrier (BBB) and can thus confirm inflammatory processes within the central nervous system (CNS). An intrathecal IgG synthesis can be observed in infectious, malignant and chronic inflammatory diseases of the CNS [5, 14, 15, 16, 17]. Pathogen‐specific antibody indices indicate a humoral immune response in the CNS which is important for the diagnosis of infectious and autoimmune CNS diseases [18]. The measles‐, rubella‐, varicella zoster‐reaction (MRZR) is one of the markers indicative for multiple sclerosis (MS) [6]. A positive MRZR is defined as a positive intrathecal response to at least two of the three viral agents [6].

We here set out to investigate age‐ and sex‐dependent effects on CSF markers [2, 4, 5, 10, 19] including effects on IgG, IgA, and IgM and IgG index. In addition, we analyzed whether there is an effect of sex and age on the antibody indices for MRZR and borrelia as well as herpes zoster virus. With our study, we aim to contribute to sex‐ and age‐directed medicine and highlight the importance of corrected reference values in CSF markers.

## 2 Methods

### 2.1 Study Design

The study was authorized by the local ethics committee of the Ruhr‐University Bochum (reg. no. 4493‐12). All CSF punctures performed in the Department of Neurology, Ruhr‐University Bochum, St. Josef Hospital Bochum and processed in the CSF laboratory between January 2012 and June 2020 were analyzed for the study (*n* = 4163). The Department of Neurology treats the whole spectrum of neurological disorders with a focus on multiple sclerosis and related disorders, neurodegenerative disorders, stroke and neuropathies [20]. Lumbar punctures in this setting are performed for defined diagnostic or therapeutic indications. The dataset was fully anonymized and did not contain information on clinical diagnoses or on whether CSF sampling had been repeated in the same individual. The study was designed to analyze age and sex‐related effects in CSF samples with largely normal CSF findings, rather than to characterize disease‐specific alterations. To approximate a cohort with largely normal CSF, we applied a purely CSF‐based exclusion strategy using the locally established reference ranges that are routinely applied in the CSF laboratory for clinical reporting. Clinical information for sample detection was not available due to anonymization of the dataset.

**FIGURE 1:**
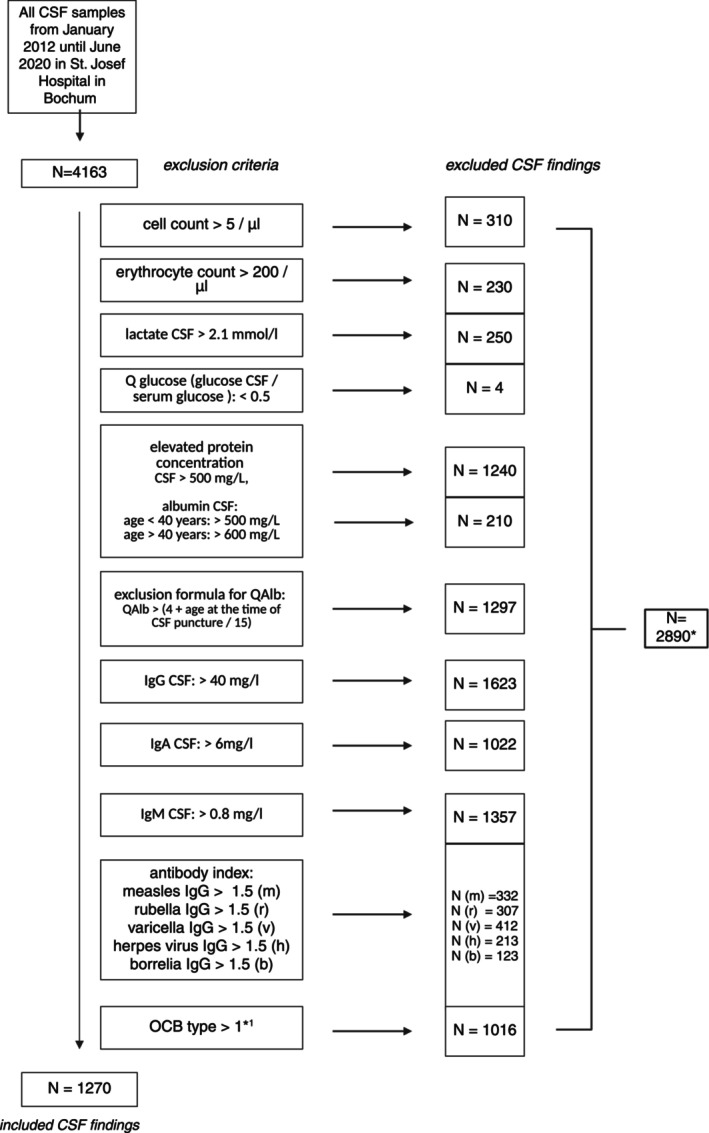
Flow chart of inclusion of CSF samples. Criteria for patient exclusion included the following: Cell count: > 5/μL, erythrocytes: > 200/μL, lactate in CSF: > 2.1 mmol/L, Q glucose: < 0.5, CSF protein in mg/L: > 500 mg/L, CSF albumin in mg/L: Up to 40 years of age > 500 mg/L, from 40 years of age > 600 mg/L. Q Albumin reference values were adjusted for age, excluding patients where applicable based on the formula Q Alb (> 4 + age at time of CSF collection/15) according to Reiber [21]. Additional exclusion parameters were IgG CSF: > 40 mg/L, IgA CSF: > 6 mg/L, IgM CSF: > 0.8 mg/L, measles IgG antibody index > 1.5, rubella IgG antibody index > 1.5, varicella IgG antibody index > 1.5, herpes IgG antibody index > 1.5, borrelia IgG antibody index > 1.5, OCB type > Type 1.*^1^ *N* = number of samples. There are overlaps between exclusion criteria and findings. *This value does not represent a sum. Three findings were without any data to CSF biomarkers (Table S1). *^1^OCB (oligoclonal bands) are detected with isoelectric focusing an immunodetection using silver staining. It is a non‐specific sign in subacute and chronic inflammatory CNS diseases (Type 1: Normal; Type 2–5: Pathological) [17].

There are already age‐adjusted reference values for Q Alb and for CSF albumin [1, 5]. For the calculation of Q Alb, the formula according to Reiber is used [5]. Although age‐adjusted reference ranges for CSF protein are frequently used in clinical practice, these were not yet established in the current neurological S1 guideline at the time the study was conducted. We therefore did not apply age‐dependent reference values for CSF protein [1]. On this basis, we excluded all CSF samples that showed any relevant CSF abnormality according to the local reference ranges (Figure 1, Table S1). In this study, we refer to biological sex‐based on genetic composition and reproductive organs [22]. Patients are categorized as male or female.

### 2.2 Laboratory

Lumbar punctures were performed after written informed consent as part of routine diagnostic work‐up under standard conditions in everyday clinical practice. CSF samples were delivered to the CSF laboratory within the hospital. There were no instrument changes during the data collection period 2012–2020. The cell count was determined manually under the microscope using a Fuchs‐Rosenthal cell chamber [23]. After cell count, CSF was centrifuged using the Hettich centrifuge Rotina 46 RS. CSF and plasma glucose, as well as lactate and CSF protein were determined using the DiaSys Respons 910. CSF glucose was measured by the hexokinase method [24]. The CSF lactate concentration was determined by using an enzymatic UV test with lactate dehydrogenase. The amount of nicotinamide adenine dinucleotide (NADH) formed by this enzymatic reaction was proportional to the lactate concentration [25]. The protein in the CSF was determined using a photometric test with pyrogallol red. Albumin, IgG, IgM, IgA in serum as well as in CSF were measured using kinetic nephelometry (IMMAGE 800 Beckmann Coulter). The CSF/serum ratios (QIgG, QIgM, QIgG, and QAlb) were calculated. For the determination of the immune status and the detection of intrathecal IgG antibodies in CSF for MRZ, borrelia and herpes virus, the immunomat VIRION/SERION was used.

### 2.3 Statistics

Analyses were performed using the statistical program IBM SPSS Statistics for Macintosh v28.0.1.1 and v29.0.2. The impact of sex on the mean marker values was assessed using the *t*‐test. Levene's test was employed for analysis of variance and in case of unequal variances, *t*‐test was adjusted according to Welch. The significance threshold was set at *p* ≤ 0.05. Effect sizes were estimated using Cohen's d with interpretation guided by Cohen's (1988) criteria: < 0.20 denotes a very small effect, 0.20–0.49 indicates a small effect, 0.50–0.79 indicates a medium effect, ≥ 0.80 represents a large effect. Additionally, employing an analysis of covariance (ANCOVA), we investigated whether the impact of sex on marker mean values persisted after controlling for age. The effect of age on the data was analyzed using a single‐factor analysis of variance according to ANOVA. If a significant effect was detected, we performed post hoc test, using Bonferroni's or Tamhane's corrected *t*‐test. The level of significance was again set at *p* ≤ 0.05. The effect size was reported as Eta‐Squared *η* ^2^. The guidelines for interpreting Cohen *η* ^2^ between two means are as follows: < 0.01 indicates a negligible or very small effect, 0.01–0.05 indicates a small effect, 0.06–0.13 indicates a medium effect, and ≥ 0.14 indicates a large effect. The relationship between age and a marker in CSF was illustrated using regression lines. The Pearson correlation coefficient (*r*) describes the strength of this correlation with the following interpretation: *r* < 0.1 indicates a negligible correlation, *r* ≥ 0.1 indicates a small correlation, *r* ≥ 0.3 indicates a moderate correlation and *r* ≥ 0.5 indicates a large correlation. Using regression analyses, regression equations were created for markers with demonstrated correlations.

## 3 Results

### 3.1 Baseline Characteristics

Our data set includes a total of 4163 CSF samples (Table 1). Of those, 1898 (45.6%) samples were from male and 2265 (54.4%) from female patients. After applying the exclusion criteria, a dataset of 1270 (30.5%) CSF findings was left, consisting of 841 (66.2%) female and 429 (33.8%) male patients with a mean age of 47.17 (SD 18.18). In total, 2890 (69.4%) were excluded because of CSF values outside the currently valid reference values. For 731 (17.6%) of the excluded results, only one exclusion criterion applied. For 470 (11.3%), two exclusion criteria applied. For 1689 (40.1%) patients, there were three or more than three exclusion criteria applied (Figure 1, Table S1.13). The number of cases for each biomarker was adjusted according to data availability, as not all markers were present in every individual dataset.

**TABLE 1:** Demographic characteristics (age, sex) of recruited patients before and after applying exclusion criteria.

	Collective before applying exclusion criteria	Collective after applying exclusion criteria
Number (*n*)	4163	1270
Male (%)	1898 (45.6)	429 (33.8)
Female (%)	2265 (54.4)	841 (66.2)
Age ± SD	50.8 (± 17.91)	47.17 (± 18.18)

*Note:* The exact sample sizes for the individual markers can be found in the Tables S2.1–S2.6 and S3.1–S3.4.

### 3.2 Effect of Sex

Significant differences were found between the biological sexes for the CSF baseline parameters CSF glucose (*d* = 0.35), CSF total protein (*d* = 0.49), CSF albumin (*d* = 0.53), serum albumin (*d* = 0.28) and Q albumin (*d* = 0.43), and for IgG in CSF (*d* = 0.34), Q IgG (*d* = 0.27), IgA in CSF (*d* = 0.42), IgA in serum (*d* = 0.24), IgM in serum (*d* = −0.37), Q IgA (*d* = 0.35), and Q IgM (*d* = 0.41) with significantly higher mean values for males than for females, except for serum IgM, which was significantly higher in females (Table 2). The statistical significance was achieved for all of the markers listed above with *p* < 0.001. The estimated effect size of sex for CSF albumin was interpreted as medium according to Cohen's d (*p* < 0.001; *d* = 0.53); whereas the effect size of sex for the other markers listed above was small (*d* = 0.24 (IgA serum) to 0.49 (CSF protein)) (Table 2). On average CSF albumin was 206.5 mg/L (SD 57.29) in males and 177.2 mg/L (SD 53.79) in females (Δ*M* = 29.34; 95% CI [22.62–36.06]). For clarity, we refer to Table 2 for the means, standard deviations, and confidence intervals.

**TABLE 2:** Effect of sex on markers in CSF.

	Sex		*t*‐test	*p*	Δ*M* [95% CI]	*d*
	Male	Female				
	*M* (SD)	*M* (SD)				
CSF marker						
CSF lactate (mmol/L)	1.42 (0.20)	1.42 (0.22)	*t*(1108) = 0.15	0.882	0.00 [−0.02–0.03]	0.01
CSF glucose (mg/dL)	66.69 (10.37)	63.07 (10.37)	*t*(1104) = 5.48	< 0.001	3.63 [2.33–4.92]	0.35
Serum glucose (mg/dL)	104.63 (29.11)	100.85 (30.03)	*t*(1004) = 1.91	0.057	3.78 [−0.11–7.67]	0.13
Q glucose (mg/dL)	1.56 (0.34)	1.61 (0.78)	*t*(1004) = −1.11	0.27	−0.05 [−0.14–0.04]	−0.07
CSF protein (mg/L)	339.83 (80.93)	300.44 (79.34)	*t*(1199) = 8.09	< 0.001	39.38 [29.83–48.94]	0.49
IgG						
CSF IgG (mg/L)	24.06 (7.31)	21.59 (7.21)	*t*(1155) = 5.48	< 0.001	2.48 [1.59–3.36]	0.34
Serum IgG (g/L)	9.92 (2.46)	10.06 (2.76)	*t*(1065) = −0.83	0.41	−0.14 [−0.48–0.20]	−0.05
Q IgG	2.52 (0.73)	2.24 (1.13)	*t*(1053) = 4.16	< 0.001	0.27 [0.14–0.40]	0.27
IgA						
CSF IgA (mg/L)	2.64 (1.25)	2.13 (1.20)	*t*(1155) = 6.79	< 0.001	0.52 [0.37–0.67]	0.42
Serum IgA (g/L)	2.17 (1.48)	1.90 (0.88)	*t*(1066) = 3.78	< 0.001	0.27 [0.13–0.42]	0.24
Q IgA	1.27 (0.41)	1.12 (0.43)	*t*(1063) = 5.47	< 0.001	0.15 [0.10–0.20]	0.35
IgM						
CSF IgM (mg/L)	0.34 (0.18)	0.35 (0.19)	*t*(1155) = −0.68	0.50	−0.01 [−0.03–0.01]	−0.04
Serum IgM (g/L)	1.01 (0.53)	1.28 (0.82)	*t*(1002.13) = −6.46	< 0.001	−0.27 [−0.35–−0.19]	−0.37
Q IgM	0.43 (0.25)	0.34 (0.21)	*t*(559.88) = 5.73	< 0.001	0.09 [0.06–0.12]	0.41
Albumin						
CSF albumin (mg/L)	206.54 (57.29)	177.20 (53.79)	*t*(1157) = 8.56	< 0.001	29.34 [22.62–36.06]	0.53
Serum albumin (g/L)	43.05 (5.25)	41.57 (5.13)	*t*(1066) = 4.39	< 0.001	1.47 [0.82–2.13]	0.28
Q albumin	4.84 (1.33)	4.28 (1.31)	*t*(1066) = 6.66	< 0.001	0.57 [0.40–0.73]	0.43
IgG Index	0.52 (0.08)	0.52 (0.18)	*t*(1053) = −0.21	0.83	0.00 [−0.02–0.02]	−0.01
IgG antibody indices						
Measles	0.99 (0.18)	1.01 (0.18)	*t*(809) = −1.30	0.19	−0.02 [−0.04–0.01]	0.10
Rubella	1.00 (0.17)	0.98 (0.15)	*t*(785) = 1.22	0.22	0.01 [−0.01–0.04]	0.09
Varicella	0.99 (0.18)	0.99 (0.16)	*t*(880) = 0.56	0.57	0.01 [−0.02–0.03]	0.04
Herpes virus	1.02 (0.21)	1.02 (0.20)	*t*(617) = 0.05	0.96	0.00 [−0.03–0.03]	0.004
Borrelia	1.12 (0.22)	1.14 (0.23)	*t*(471) = −1.07	0.29	−0.02 [−0.07–0.02]	−0.10

*Note:* The effect of sex on markers in CSF was examined, with male and female mean values along with their corresponding standard deviations (SD) tabulated. A *t*‐test was conducted to determine the significance of the sex‐based differences, with the level of significance set at *p* ≤ 0.05. Cohen's d was utilized as a measure of effect size, enabling the assessment of the statistical significance between male and female mean values. Cohen's (1988) guidelines were employed for interpretation, categorizing effect size as follow: < 0.20 = “none/very small”, 0.20–0.49 = “small”, 0.50–0.79 = “medium”, ≥ 0.80 = “large”.

The effect of sex on all aforementioned markers persisted despite taking age into account as a covariant (Table S5). No significant effect of sex was observed for CSF lactate, serum glucose, Q glucose as well as for IgM in CSF and IgG in serum, the IgG index and the antibody indices for measles, rubella, varicella, herpes virus and borrelia (Table 2).

### 3.3 Effect of Age

**FIGURE 2:**
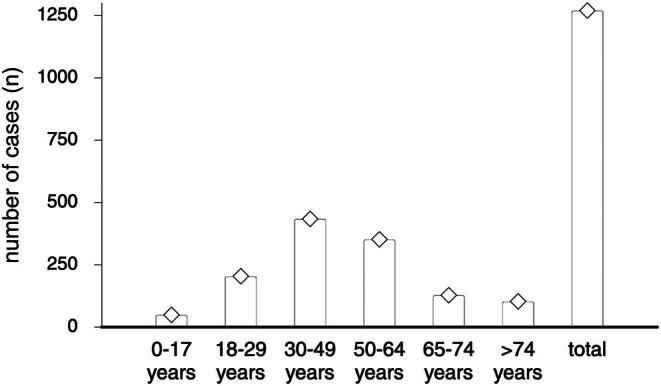
Distribution of age. Patients included in the statistical analysis for the effect of age were divided into six age groups.

**FIGURE 3:**
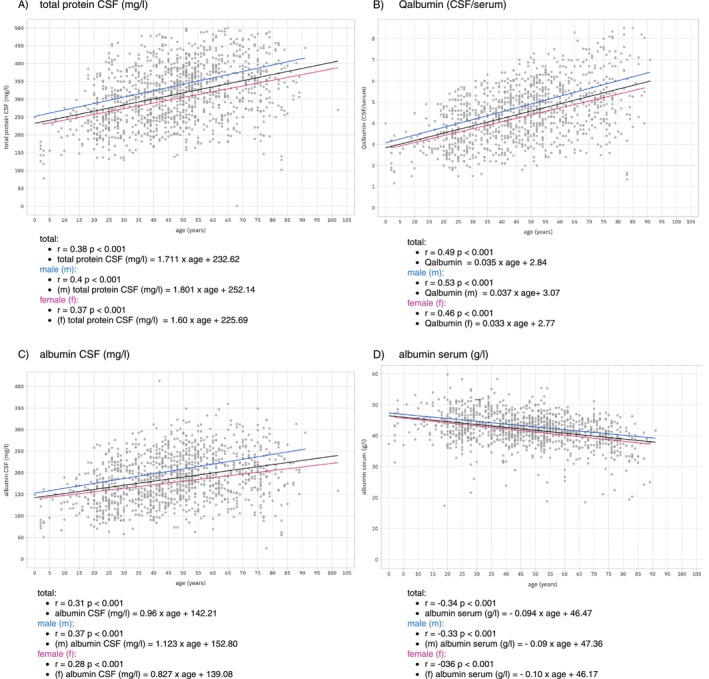
Linear regressions and regression equations for CSF protein and albumin, serum albumin and Q albumin. Linear regression analysis of age and cerebrospinal fluid (CSF) concentrations of total protein (mg/L) (A) and albumin (mg/L) (C) and albumin in serum (g/L) (D) and Q albumin (albumin CSF/albumin serum) (B) in total (black), in female (pink) and male patients (blue). For all illustrated markers, the regression lines for men are above those for woman. For total protein in CSF, albumin in CSF and Q albumin the values increase with age. For serum albumin, the values decrease with age. Pearson correlation coefficient (*r*) describes the strength of this correlation with the following interpretation: *R* < 0.1 indicates a negligible correlation, *r* ≥ 0.1 indicates a small correlation, *r* ≥ 0.3 indicates a moderate correlation and *r* ≥ 0.5 indicates a large correlation. *p*‐value describes the significance at a significance level of 5%. Based on regression analyzes for the respective markers, age‐ and sex‐adjusted regression equations can be created by using the following formula: CSF marker (X) = unstandardized coefficient of age × age + constant unstandardized coefficient (Table S7). The tables of the regression analysis with unstandardized coefficients for creating the equation can found in the Table S7.

The patients were divided into 6 age groups (Figure 2, Tables S2 and S3 (S2.3, S3.2)). The two groups between 30 and 49 years (*n* = 434) and 50–64 years (*n* = 352) represent the largest number of the cohort analyzed. A significant effect of age was found in 17 out of 18 analyzed markers (*p* < 0.001) (Table 3). This effect was strongest for CSF protein and QAlb (Table 3). For CSF protein a significant effect of age can be objectified according to Cohen's *η* ^2^ (*p* > 0.020; *η* ^2^ = 0.16); a significant effect of age was also detected for QAlb (*p* < 0.001; *η* ^2^ = 0.23) (Figure 3A,B). The correlation between age and QAlb and CSF protein, as assessed by Pearson's correlation coefficient (*r*), was regarded as moderate (Figure 3A,B).

**TABLE 3:** Effect of age on the markers in the cerebrospinal fluid.

	Age group						ANOVA	*p*	*η* ^2^ [95% CI]
	0–17 years	18–29 years	30–49 years	50–64 years	64–74 years	≥ 75 years			
	M (SD)	M (SD)	M (SD)	M (SD)	M (SD)	M (SD)			
CSF marker									
CSF lactate (mmol/L)	1.29 (0.21)	1.33 (0.19)	1.40 (0.20)	1.46 (0.20)	1.50 (0.22)	1.52 (0.24)	*F* (5, 1104) = 21.93	< 0.001	0.09 [0.06–0.12]
CSF glucose (mg/dL)	58.05 (6.34)	62.00 (7.14)	63.62 (8.30)	65.71 (12.33)	66.16 (13.07)	67.73 (13.65)	*F* (5, 257) = 12.17	< 0.001	0.04 [0.02–0.06]
Serum glucose (mg/dL)	85.56 (11.07)	91.22 (18.92)	97.94 (23.70)	108.76 (35.43)	115.32 (38.88)	110.64 (29.58)	*F* (5, 247.90) = 24.74	< 0.001	0.08 [0.05–0.11]
Q glucose (mg/dL)	1.47 (0.16)	1.48 (0.29)	1.54 (0.31)	1.70 (1.16)	1.73 (0.43)	1.64 (0.39)	F (5, 249.22) = 9.39	< 0.001	0.02 [0.00–0.04]
CSF protein (mg/L)	206.78 (54.10)	269.50 (69.89)	308.04 (74.97)	340.20 (73.82)	345.71 (86.09)	339.81 (84.93)	F (5, 265.48) = 59.98	< 0.001	0.16 [0.12–0.19]
IgG									
CSF IgG (mg/L)	15.80 (5.16)	20.61 (7.31)	22.71 (6.97)	23.36 (6.95)	23.04 (7.46)	24.39 (8.38)	*F* (5, 280.96) = 19.54	< 0.001	0.05 [0.03–0.08]
Serum IgG (g/L)	10.60 (2.40)	10.80 (2.33)	10.46 (2.70)	9.42 (2.58)	9.02 (2.29)	9.34 (3.05)	*F* (5, 1061) = 13.40	< 0.001	0.06 [0.03–0.08]
Q IgG	1.58 (0.41)	1.94 (0.59)	2.22 (0.66)	2.53 (0.70)	2.79 (2.24)	2.80 (0.89)	*F* (5, 256.11) = 46.93	< 0.001	0.10 [0.07–0.13]
IgA									
CSF IgA (mg/L)	0.97 (0.57)	1.87 (1.06)	2.24 (1.14)	2.62 (1.26)	2.66 (1.30)	2.68 (1.39)	F (5, 299.30) = 56.86	< 0.001	0.10 [0.07–0.13]
Serum IgA (g/L)	1.19 (0.65)	1.89 (0.91)	2.00 (0.85)	2.13 (1.66)	2.06 (0.80)	2.05 (0.90)	*F* (5, 1062) = 5.83	< 0.001	0.03 [0.01–0.04]
Q IgA	0.82 (0.29)	0.98 (0.35)	1.13 (0.38)	1.30 (0.42)	1.30 (0.40)	1.39 (0.56)	*F* (5, 258.77) = 33.04	< 0.001	0.12 [0.08–0.15]
IgM									
CSF IgM (mg/L)	0.22 (0.12)	0.33 (0.18)	0.36 (0.19)	0.35 (0.18)	0.33 (0.18)	0.39 (0.20)	*F* (5, 287.40) = 13.03	< 0.001	0.03 [0.01–0.05]
Serum IgM (g/L)	1.33 (0.52)	1.29 (0.55)	1.30 (0.71)	1.08 (0.92)	1.06 (0.84)	0.93 (0.49)	*F* (5, 1062) = 6.76	< 0.001	0.03 [0.01–0.05]
Q IgM	0.23 (0.22)	0.29 (0.16)	0.34 (0.20)	0.40 (0.22)	0.40 (0.23)	0.53 (0.34)	*F* (5, 227.40) = 15.84	< 0.001	0.09 [0.06–0.12]
Albumin									
CSF albumin (mg/L)	131.72 (36.77)	159.10 (49.01)	185.98 (52.91)	204.51 (53.46)	205.70 (55.58)	199.52 (64.99)	*F* (5, 282.84) = 41.90	< 0.001	0.12 [0.08–0.15]
Serum albumin (g/L)	44.56 (4.95)	43.54 (5.51)	43.05 (4.51)	41.81 (4.75)	40.36 (4.83)	37.16 (5.31)	*F* (5, 1062) = 31.06	< 0.001	0.13 [0.09–0.16]
Q Albumin	2.92 (0.81)	3.67 (1.03)	4.27 (1.10)	4.88 (1.16)	5.15 (1.32)	5.49 (1.66)	*F* (5, 265.05) = 71.29	< 0.001	0.23 [0.18–0.27]
IgG Index	0.53 (0.06)	0.53 (0.07)	0.52 (0.08)	0.52 (0.08)	0.54 (0.43)	0.50 (0.07)	*F* (5, 1049) = 0.99	0.42	0.00 [0.00–0.01]

*Note:* Presentation of the mean values with corresponding standard deviations within the individual age groups. Data was divided into six age groups. The comparison of the mean values between the six age groups is carried out using a single‐factor analysis of variance (ANOVA). If the *p*‐value of the respective factor was below the selected significance level (< 0.05), there was a significant difference between at least two groups represented by this factor. The effect size is presented as Eta‐Squared *η* ^2^. The guidelines for the interpretation of Eta‐Squared *η* ^2^ to assess the statistical significance of a difference between two mean values are as follows: < 0.01 = “negligible”; 0.01–0.05 = “small”; 0.06–0.13 = “medium”, ≥ 0.14 = “large”.

### 3.4 Comparison of Age and Sex Effects on Markers in CSF

Throughout almost all markers in CSF either a significant effect of age only on the markers or a significant effect of sex and age on the markers was shown. For CSF lactate, Q glucose, and IgM in CSF there were no significant effects of sex within our dataset whereas the effect of age on CSF lactate is medium and for Q glucose and for IgM in CSF a small effect of age could be shown (Tables 2 and 3). CSF lactate showed a moderate correlation with age (*r* = 0.31). CSF lactate increased significantly with increasing age, whereas the correlation of age and IgM in CSF (*r* = 0.09), or age and Q glucose (*r* = 0.13) was considered small or negligible (Figure 5C, Table S7).

**FIGURE 4:**
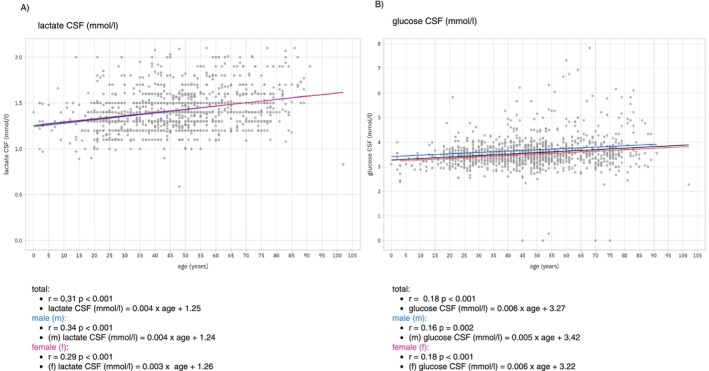
Linear regressions and regression equations for CSF lactate and glucose. Linear regression analysis of age and cerebrospinal fluid (CSF) concentrations of lactate (mmol/L) (A) and glucose (mmol/L) (B) in total (black) and in female (pink) and male patients (blue). For lactate in CSF (A) there is no significant effect of sex (see Table 2) as evidenced by nearly identical regression lines for men and woman. Lactate in CSF increases with age. The regression line for glucose (B) is higher for man than for woman. Glucose in CSF increases with age. Pearson correlation coefficient (*r*) describes the strength of this correlation with the following interpretation: *R* < 0.1 indicates a negligible correlation, *r* ≥ 0.1 indicates a small correlation, *r* ≥ 0.3 indicates a moderate correlation and *r* ≥ 0.5 indicates a large correlation. *p*‐value describes the significance at a significance level of 5%. Based on regression analysis for the respective markers, age‐ and sex‐adjusted regression equations can be created by using the following formula: CSF marker (X) = unstandardized coefficient of age × age + constant unstandardized coefficient (Table S7). The tables of the regression analysis with unstandardized coefficients for creating the equation can found in the Table S7.

For CSF glucose and IgG in CSF the effect sizes for both sex and age on CSF glucose and IgG in CSF were small (Tables 2 and 3). The correlation between age and CSF glucose was considered small (*r* = 0.18), with a tendency for CSF glucose levels to increase with age (Figure 4B). For CSF albumin the effect size occurred to be medium for both age and sex. CSF albumin increased with age and male sex.

For serum albumin, the effect of sex was small whereas the effect of age was medium. Serum albumin decreased with growing age; the negative correlation is considered as moderate (Tables 2 and 3, Table S7).

**FIGURE 5:**
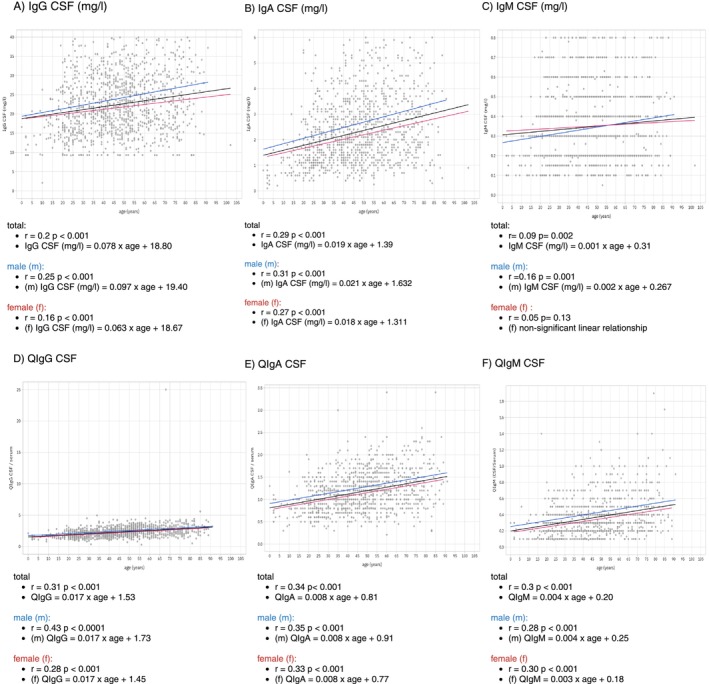
Linear Regressions and regression equations for Immunoglobulin IgA, IgG, and IgM and QIgG, QIgA, QIgM. Linear regression analysis of age and cerebrospinal fluid (CSF) concentrations of IgG (mg/L) (A), IgA (mg/L) (B), IgM (mg/L) (C) and QIgG (D), QIgA (E) and QIgM (F) in total (black) and in female (pink) and male patients (blue). Pearson correlation coefficient (*r*) describes the strength of this correlation with the following interpretation: (*r* < 0,1 negligible correlation, *r* ≥ 0,1 small correlation, *r* ≥ 0,3 moderate correlation and *r* ≥ 0,5 large correlation.) *p*‐value describes the significance at a significance level of 5%. Based on regression analysis for the respective markers, age‐ and sex‐adjusted regression equations were created according to the formula: CSF marker (X) = unstandardized coefficient of age x age + constant unstandardized coefficient (Table S7). The tables of the regression analysis with unstandardized coefficients for creating the equation can found in the Table S7. For all immunoglobulins except IgM in CSF, there is a significant effect of sex and age on the marker, with higher values in males and increasing values with age. This is also illustrated by the regressions and equations where the linear relationship between IgM and age is negligible without considering sex (males). For females, there is not a significant linear correlation for IgM in CSF.

A small effect of sex was observed for the quotients of immunoglobulins QIgG, QIgM, and QIgA as well as CSF IgA (Table 2). In contrast, analyzes for age revealed a medium to strong effect of age on these markers (Table 3, Figure 5A–F). A small effect of sex was observed for QAlb, while a large effect of age was demonstrated for QAlb. Similarly, for CSF protein, a small sex effect and an equally large age effect were observed. Consequently, the most significant effect for both sex and for age was found for CSF protein and QAlb. On average, the values were significantly higher in older and in male patients (Figure 3A,B, Tables 2 and 3).

For CSF IgG index as well as antibody indices for rubella, herpes, and borrelia virus, no significant effect of age or sex could be documented (Tables 2 and 3, Tables [Link], [Link], [Link], [Link]).

## 4 Discussion

In our study we detected significant effects of sex and age in our predominantly Caucasian cohort for almost all common CSF markers. Additionally, we were able to formulate sex‐ and age‐dependent regression equations for a variety of markers with a relevant effect of sex or age, which may serve as guidance for sex‐ and age‐adjusted reference ranges.

In neurological diagnostics and treatment planning, even modest sex‐ or age‐related differences can be of diagnostic importance (e.g., in radiculo‐neuritis). However, the statistical significance of a marker alone does not establish causality nor imply clinical relevance. To properly assess such clinical value, diagnosis‐related data are required. Ideally, such effects should be examined in “healthy subjects” to minimize potential confounders.

In practice, however, this is problematic: lumbar puncture, as an invasive procedure, cannot be ethically justified without clinical indication due to associated risks (infection, post‐puncture headache, hygroma) [1].

Therefore, future studies based on diagnosis‐ and therapy‐related data are essential, for example by comparing different patient groups or by conducting longitudinal analyses within defined diagnostic cohorts.

### 4.1 CSF Protein and QAlb

The strongest effect of age could be demonstrated for CSF protein and QAlb with a positive correlation between age and either CSF protein or QAlb, as previously described. Additionally, there was an effect of biological sex for both markers. According to our results, CSF protein and QAlb are significantly higher in older male patients than in elderly women. Despite possible interlaboratory analytical variability for different studies, our study goes in line with the results of previous studies by Castellazzi et al., Skillbäck et al., and Parrado et al. and Seyfert et al. [10, 19, 26, 27, 28, 29], and therefore supports the thesis of age and sex dependent QAlb and CSF protein levels. Parrado et al. observed similar associations in several hundred healthy individuals from a large, anonymized cohort, suggesting that the absence of diagnostic information in our dataset likely has a limited impact on QAlb validity. QAlb and CSF protein are both essential markers for evaluating the blood–brain barrier (BBB) and the blood‐CSF‐barrier (BCSFB) function, hence important for the diagnosis of immune‐mediated neuropathies, neuro inflammatory, and neurodegenerative diseases [5, 10, 28, 30, 31, 32].

CSF protein is a frequently used and often unspecific marker in the diagnosis of neurological diseases. Although CSF protein values are interpreted age‐adapted in many clinical laboratories, there are guidelines such as the S1 guideline of the German Society for Neurology which do not yet take age‐ nor sex‐adjusted reference values into account [1, 12, 28, 33]. Using the fixed cut‐off value (500 mg/L) is a potential danger for misdiagnosing blood‐CSF‐barrier dysfunction among younger female individuals, with a risk of false negative diagnosis without age‐ and sex‐adjusted reference values in elderly male individuals [28]. At the same time, older male patients with a well‐functioning blood‐CSF barrier show a falsely elevated total CSF protein level, potentially leading to false positive results. A recently published study by Seeliger et al. compares the diagnostic value of QAlb and CSF protein in relation to immune‐mediated neuropathies, highlighting the central importance of QAlb as the most reliable marker for evaluating blood‐CSF barrier function, particularly in younger patients with immune‐mediated neuropathies [28]. While CSF protein measurements are readily available and cost‐efficient, they are subject to fluctuations due to the dependency of the absolute CSF protein concentration, age and CSF flow rate. Additionally, intrathecal antibody synthesis can also affect total CSF protein levels [5, 34]. Therefore, measuring the absolute protein concentration in the CSF can yield normal results even if there is a disruption in the blood‐CSF barrier, complicating the interpretation of findings [35, 36].

QAlb, introduced in 1977 [16], is age‐adjusted and independent of intrathecal protein synthesis, resulting in a highly reliable tool for assessing BBB integrity [5, 10, 26, 30, 36]. It is altered in multiple neurodegenerative and inflammatory diseases [30, 31]. Current reference ranges are usually age‐adjusted but do not consider sex as a potential confounder, although our data, in line with previous reports, confirm significantly higher mean QAlb values in male patients [10, 13, 26, 27].

Reiber's diffusion‐flow model proposes that an extended CSF flow path (e.g., in taller individuals) or reduced flow rate increases QAlb and CSF protein, but a direct correlation with body height has not yet been demonstrated [34, 37, 38]. Our dataset lacks height, weight, and BMI, yet prior studies indicate that BMI influences CSF markers and is associated with increased QAlb, while sex remains an independent predictor even after BMI adjustment [27, 29]. Thus, sex‐ and age‐dependent QAlb levels and thereby BBB permeability are highly relevant not only for diagnosing neurological diseases but also for CNS pharmacotherapy, where sex‐specific pharmacological properties must be considered.

In our cohort, we confirm the sex‐dependency of QAlb across all age groups but cannot account for additional determinants such as BMI, vascular, hormonal, or inflammatory factors due to missing variables. Given that only few studies have examined the combined effects of sex, age, and barrier function, our findings highlight the need to future studies incorporating detailed anthropometric and clinical data to refine sex‐ and age‐specific reference values and clarify their diagnostic and therapeutic implications.

### 4.2 IgG Index

The IgG index is used to differentiate intrathecal IgG synthesis from a dysfunction of the BBB and can thus confirm inflammatory processes within the CNS that can be observed in infectious, malignant, and chronic inflammatory diseases of the CNS [5, 14, 15, 16, 17].

The IgG index, alongside oligoclonal IgG, is one of the most sensitive diagnostic markers for detecting intrathecal immunoglobulin synthesis as a sign of CNS inflammation such as in MS [39]. Although not specifically included in the international diagnostic criteria for MS, that is, McDonald criteria, the IgG index is considered a cost‐effective parameter and appears to be highly indicative of OCB positivity [39]. The dataset analyzed here showed no influence of age or sex on the IgG index, deeming it a tool independent of age and sex.

### 4.3 MRZ Reaction and Antibody Indices (AI) for Herpes Virus and Borrelia, IgA, IgM, IgG

Immunoglobulins in the CSF provide important information about the immunological status of the CNS [35]. Immunological response patterns are utilized for diagnostic purposes [17]. Pathogen‐specific AI reflect a humoral immune response within the CNS, which is important for the diagnosis of infectious and autoimmune CNS diseases [18]. Among these, the MRZR stands out as one of the most important supporting markers to diagnose MS [6].

In our study, there was no significant statistical effect of age or sex on the AI for the MRZR, borrelia, or herpes zoster virus. However, for IgA, IgM, and IgG in CSF, a statistical effect was evident for both age and sex, although the effect size was small.

The validity of these results needs to be critically assessed. While it is known that immunosuppressive therapies can affect intrathecal immunoglobulin synthesis, our dataset does not provide information about ongoing therapies. Previous studies have shown that immunosuppressive therapies can reduce the intrathecal synthesis of immunoglobulins as well as the AI of specific antibodies [7, 40, 41, 42]. We excluded antibody indices considered pathological from the statistical analysis using our exclusion criteria. However, we cannot assess whether the findings with normal AI were indeed normative due to ongoing therapy. Therefore, for a reliable interpretation of investigation on AI and immunoglobulins in the CSF, it is essential to have information regarding diagnosis and ongoing therapies.

### 4.4 CSF Glucose and Lactate

There are only a few studies with conflicting findings regarding the examination of age‐ and sex effects on CSF lactate and CSF glucose, despite these being fundamental markers routinely analyzed in nearly every lumbar puncture [2, 43]. In our study, men exhibited significantly higher mean CSF glucose levels compared to women. Thus, our study is one of the first to demonstrate an effect of sex on CSF glucose levels. Furthermore, we identified an association between age and CSF glucose levels, demonstrating an increase in CSF glucose levels with advancing age. This finding aligns with the conclusions drawn by Leen et al., who investigated age‐dependent reference values for CSF glucose and lactate [2].

When energy‐consuming diseases are present, there is a decline in CSF glucose levels. For example, CSF glucose levels are decreased in patients with bacterial meningitis, leptomeningeal carcinomatosis or neurosarcoidosis, typically in combination with other abnormal CSF parameters [2, 43, 44]. The current reference values may pose the risk of imprecise interpretation of CSF glucose leading to false‐negative values in men and older patient groups. This can potentially result in delayed initiation of treatment, which, for example, in bacterial meningitis, would significantly worsen the outcome for this patient group [45].

Moreover, CSF glucose levels are influenced by serum glucose metabolism [43]. Hence, leveraging the Q glucose ratio facilitates a more accurate interpretation of CSF glucose levels. Our study also reveals a significant age‐related effect on Q glucose ratio, indicating a tendency for increased Q glucose levels with advancing age. Similar to Leen's study, our investigation lacks additional information on diseases or ongoing therapies affecting glucose metabolism. Interestingly, a study published in 2023, albeit with a markedly smaller sample size, incorporated glucose metabolism into their analysis and found no age or sex effect on glucose levels [43]. This suggests that glucose metabolism may serve as a confounding factor in assessing age and sex dependence. Future studies on age‐dependency and sex‐dependency of CSF glucose should therefore consider various factors influencing glucose metabolism such as drugs, nutrition, and diabetes mellitus.

Elevated levels of CSF lactate can occur in various conditions, such as subarachnoid hemorrhage, bacterial meningitis, cerebral hypoxia, and status epilepticus [2, 46, 47, 48]. For CSF lactate, our study demonstrates that sex does not have an impact, while age has a significant increasing effect on CSF lactate levels. This finding supports previous findings and is already taken into account in the current S1 guideline [1, 2, 43, 48].

There are several limitations of the current study which need to be taken into consideration. First, our dataset predominantly included Caucasians. Previous studies have already demonstrated an effect of ethnicity on the IgG index in cerebrospinal fluid, with a higher IgG index observed in African Americans compared to Caucasians [7, 39]. Additionally, the age distribution of our dataset is unbalanced, with the majority of participants being in middle age groups. The applied covariance analysis (ANCOVA) can help to control for the effect of the covariate age and thus minimize some of the biases caused by an unbalanced age distribution, especially since younger populations are less likely to undergo diagnostic lumbar puncture—except for suspected MS. Careful planning of further studies with a balanced age distribution and a study cohort of mixed ethnicity can help further maximize the generalizability of the results. One important limitation is the missing information regarding personal data (e.g., height), comorbidities and indications for lumbar puncture. Hence, neuroinflammatory and neurodegenerative diseases as well as CNS penetrating immunotherapies might have been a significant biasing factor. Nevertheless, the observed effects of age and sex on CSF parameters in our cohort are partly consistent with previous reports in healthy volunteers, which supports the validity of our cohort defined by normal routine CSF parameters.

In summary, our study highlights that age and sex have an effect on the majority of the markers examined in CSF. This study reinforces some previous studies and places the statistical effects in the context of neurological clinical practice. It thus underscores the importance of age‐ and sex‐adjusted reference values for the interpretation of CSF markers in clinical practice. To further improve the validity of the results, including diagnoses and ongoing therapies in the evaluation would be an important enhancement for future studies. With our study, we aim to contribute to sex‐ and age‐adapted medicine. In our study, we refer to biological sex. Gender refers to individuals' identities, which are influenced by cultural norms and societal roles [9, 22]. This affects lifestyle and thus the risk of diseases, so in addition to biological sex, individual identity, defined as gender, should also be considered in the planning of future studies and the interpretation of results. The importance of gender‐ and age‐adapted medicine can be illustrated by the example of MS. Here we already know that susceptibility to the disease and its progression significantly depend not only on sex and age, but also on fertility, pregnancy and ethnicity [15, 49, 50, 51]. Diagnostic tools and therapy options must be adapted considering ensuring the best possible and low‐risk diagnosis and treatment of individuals with neurological conditions.

## Author Contributions

I.‐S.H., S.F., R.G., A.H. acquired patient's samples. I.‐S.H. and punkt05 statistics in Düsseldorf made the figures. I.‐S.H. wrote the first draft of the manuscript. S.F., R.G., and A.H. critically revised the manuscript. A.H. designed and supervised the study. All authors read and approved the final manuscript.

## Funding

The authors have nothing to report.

## Conflicts of Interest

The authors declare no conflicts of interest.

## Supporting information


**Table S1:** Overview of exclusion frequencies for each cerebrospinal (CSF) marker based on predefined exclusion criteria. The different tables list the number and percentage of participants excluded due to values outside predefined criteria.

**Table S2:** Summary of cohort characteristics after applying exclusion criteria, including age distribution, age group sizes, and number of CSF samples analyzed per biomarker.

**Table S3:** Age and sex distribution of the study cohort after application of exclusion criteria. The table includes all 1270 individuals meeting inclusion criteria. For some CSF parameters, cohort size may vary due to missing data in individual laboratory reports. Distributions of age and sex are shown for each parameter after applying exclusion criteria.

**Table S4:** acn370307‐sup‐0004‐DataS4.xlsx. *T*‐test results after application of exclusion criteria to assess the effect of sex on each cerebrospinal fluid (CSF) biomarker. Statistically significant effects are highlighted in green.

**Table S5:** Analysis of covariance (ANCOVA) assessing the effect of sex considering age (in years) as a confounder on cerebrospinal fluid (CSF) biomarkers. The table summarizes the statistical results for sex and age‐dependent effects. Statistically significant effects are highlighted in green.

**Table S6:** Pairwise *t*‐test comparisons examining the effect of age on cerebrospinal fluid (CSF) biomarkers across age groups. Bonferroni or Tamhane corrections were applied depending on the assumption of equal variances.

**Table S7:** Regression analysis to model age‐dependent reference values for individual cerebrospinal fluid (CSF) biomarkers. Relationships between cerebrospinal fluid (CSF) biomarkers and age were evaluated using Pearson correlation and linear regression. Unstandardized regression coefficients B describe the age‐dependent change in each biomarker.

## Data Availability

All data are available from the corresponding author AH upon reasonable request.
